# Frequency of Cardiovascular Manifestation in Patients With Hyperthyroidism

**DOI:** 10.7759/cureus.12839

**Published:** 2021-01-21

**Authors:** Naresh Kumar Khurana, Suresh Kumar, Sameet Kumar, Pardeep Kumar, Amber Rizwan

**Affiliations:** 1 Cardiology, Central Park Medical College, Lahore, PAK; 2 Internal Medicine, Bolan University of Medical & Health Sciences, Quetta, PAK; 3 Internal Medicine, Chandka Medical College, Karachi, PAK; 4 Medicine, Jinnah Sindh Medical University, Karachi, PAK; 5 Family Medicine, Jinnah Post Graduate Medical Center, Karachi, PAK

**Keywords:** hyperthyroidism, cardiovascular symptoms

## Abstract

Introduction

Cardiovascular manifestations are very common in hyperthyroidism. Various cardiovascular symptoms such as palpitations, exercise intolerance, dyspnea, angina, edema, and congestive heart failure are commonly reported in hyperthyroidism. In this study, we determine the frequency of cardiovascular signs, symptoms, and various conduction disorders associated with hyperthyroidism.

Methodology

This cross-sectional, observational study was conducted in the cardiology department of a tertiary care hospital in Pakistan in close association with the internal medicine department from August 2019 to December 2019. A total of 100 hyperthyroid patients confirmed based on thyroid stimulating hormone (TSH, also known as thyrotropin), free T_4_ (FT_4_; thyroxine), and free T_3_ (FT_3_; triiodothyronine) were enrolled in the study.

Results

The most common cardiovascular symptom in this study was palpitations identified in 72% of the participants, followed by breathlessness in 41% of the participants. The most common cardiovascular sign was a pulse rate of more than 100 beats per minute found in 72% of the participants. The most common abnormality in electrocardiogram (ECG) was sinus tachycardia in 39% of the participants, followed by atrial fibrillation in 22% of the participants. In echocardiography, 5% of the participants had systolic dysfunction.

Conclusion

In this study, cardiovascular signs, symptoms, ECG, and echo changes were very frequent in hyperthyroidism. Management of hyperthyroidism should include routine ECG and echo testing, and cardiologists should be involved in thorough cardiovascular examination.

## Introduction

Hyperthyroidism is defined as excess concentration of thyroid hormones in the body due to either increased synthesis of the thyroid hormone, increased release of preformed thyroid hormones, or from endogenous or exogenous extrathyroidal sources [1]. Hyperthyroidism is very prevalent worldwide. According to a 2002 study, Pakistan reported a prevalence of 5.1% for hyperthyroidism and 5.8% for clinical hyperthyroidism [2].

Clinical symptoms of hyperthyroidism depend upon various factors including patient’s age, sex, presence of other diseases, duration of the disease, and cause of the disease. Older patients exhibit fewer and less severe symptoms but may end up with more cardiovascular complications [3-5]. Various cardiovascular symptoms such as palpitations, exercise intolerance, dyspnea, angina, edema, and congestive heart failure are commonly present in patients with hyperthyroidism [6,7]. Atrial fibrillation is reported in 10-25% of hyperthyroid patients and may cause further complications [8].

Despite hyperthyroidism being very prevalent in Pakistan, there is very limited data available in Pakistan, particularly related to cardiovascular symptoms and complications. In this study, we determine the frequency of cardiovascular signs, symptoms, conduction disorders, and arrhythmias in hyperthyroidism.

## Materials and methods

This cross-sectional, observational study was conducted in the cardiology department of a tertiary care hospital in Pakistan in close association with the internal medicine department from August 2019 to December 2019. Ethical review board approval was taken from the Bolan University of Medical Health Sciences before the start of the study. A total of 100 patients under treatment of hyperthyroid confirmed based on thyroid stimulating hormone (TSH, also known as thyrotropin), free T_4_ (FT_4_; thyroxine) and free T_3_ (FT_3_; triiodothyronine) were enrolled in the study. Patients with hypertension, diabetes mellitus, congestive heart failure, and coronary artery disease were excluded from study.

After obtaining informed consent, patient history was taken for cardiovascular symptoms such as palpitations, breathlessness, chest pain, and edema. Patient pulse and standing blood pressure were recorded via digital instruments. Cardiovascular auscultation was done to identify any abnormal heart sound. Chest X-ray (cardiothoracic ratio above 0.5) was done to identify cardiomegaly. Electrocardiogram (ECG) and echocardiography was done to identify various abnormalities.

Statistical analysis was done using the Statistical Package of Social Sciences (SPSS) version 21.0 (IBM Corporation, Armonk, New York, USA). Continuous variables were presented as means and standard deviations, while categorical variables were presented as percentages and frequencies.

## Results

The mean age of the participants was 39 ± 12 years. There were 21 (21%) males and 79 (79%) females in the study. The mean duration of diagnosis of hyperthyroidism was 4 ± 2 years. Mean TSH level was 0.091 ± 0.09 mU/L. Mean FT_4_ was 2.22 ± 0.31 ng/dL. Mean FT_3_ was 4.51 ± 0.25 ng/dL.

The most common symptom in this study was palpitations (72%), followed by breathlessness (41%). The most common sign was tachycardia (pulse rate of more than 100 beats per minute) identified in 72% of the participants (Table 1).

**Table 1:** Cardiovascular signs and symptoms in hyperthyroid patients.

Signs and symptoms	Percentage
Edema (%)	14
Palpitations (%)	72
Breathlessness (%)	41
Chest pain (%)	06
Number of participants with systolic blood pressure greater than 130 mmHg (%)	41
Number of participants with diastolic blood pressure greater than 80 mmHg (%)	07
Number of participants with pulse rate greater than 100 beats per minute (%)	72
Loud pulmonary (P2) sound	03
Other sounds	21

In chest X-ray, cardiomegaly was present in 10% of the participants. The most common finding in ECG was sinus tachycardia (39%), followed by atrial fibrillation (22%). In echocardiography, 5% of the participants had systolic dysfunction (Table 2).

**Table 2:** Cardiovascular diagnostic findings in hyperthyroid patients. ECG, electrocardiogram

Diagnostic findings	Percentage
Chest X-ray findings	
Cardiomegaly	10
ECG findings	
Sinus tachycardia	39
Atrial fibrillation	22
Right bundle branch block	04
Left bundle branch block	04
Right ventricular hypertrophy	06
Left ventricular hypertrophy	06
Supraventricular tachycardia	02
Echocardiography findings	
Systolic dysfunction	05
Diastolic dysfunction	01
Regurgitant lesions	08
Pulmonary hypertension	02

## Discussion

Various direct and indirect mechanisms are responsible for the influence of thyroid hormone on the heart and cardiovascular system [9]. Thyroid hormones influences myocytes by upregulating alpha (α)-chain, but downregulates beta (β)-chain [10]. It may also influence sarco/endoplasmic reticulum, which may increase the rate of calcium uptake during diastole [11,12]. Thyroid hormone also directly acts on ion channels such as Na/K-ATPase, Na/Ca++ exchanger, and some voltage-gated K channels, hence affecting myocardial and vascular functions [13,14]. Other than the cellular impact of thyroid hormone, it also influences the hemodynamic balance in the body by its direct effects on the heart and blood vessels [13]. Thyroid hormones may cause rapid use of oxygen by the body, increased production of metabolic products, and relaxation of arterial smooth muscle, which may lead to peripheral vasodilation [13].

Cardiac symptoms seen in hyperthyroidism either may be due to the effect of increased sympathoadrenal activity or due to the direct effect of thyroid hormones on the heart [15]. In this study, the most common cardiac symptoms were palpitation, followed by breathlessness. Kandan et al. reported in their study that the most common cardiovascular manifestation was palpitation (78%), followed by dyspnea/breathlessness (26%) and chest pain (4%) [16]. In this study, tachycardia (pulse rate greater than 100 beats per minute) was found in 72% of the participants, which was comparable to the studies of Kandan et al. and Zargar et al. [16,17].

In our study, atrial fibrillation was seen in 22% of the participants, which was comparable to the study conducted by Kandan et al., which reported 28% prevalence of atrial fibrillation [16]. However, the prevalence in our study was much higher than that reported by a Saudi study conducted by Zargar et al., which reported a prevalence of 8.9% [17]. Low serum TSH is an independent risk factor for development of atrial fibrillation [18]. Atrial fibrillation has been proven to cause more mortality and morbidity due to embolic events in patients with hyperthyroidism [19]. ECG findings such as P-maximum and P-wave dispersion are important identifiers of paroxysmal atrial fibrillation [20]. In this study, systolic dysfunction was seen in 5% of the participants, which was comparable to that reported by Mercé et al. (3%) [21]. Kandan et al. reported much higher prevalence (18%) of systolic dysfunction in their study [16].

To the best of our knowledge, this is the first local study to identify cardiovascular manifestation in hyperthyroid patients. The study has its limitations as well. As all the participants were from one institute, the sample size was limited and less diverse. In addition, as it was cross-sectional study, long-term impact of cardiovascular manifestations cannot be determined.

## Conclusions

In this study, cardiovascular manifestations such as tachycardia, breathlessness, and atrial fibrillation were very prevalent in patients with hyperthyroidism. It is important to identify cardiovascular manifestations early in patients and manage them appropriately to reduce mortality and morbidity risk. Cardiologists should be involved in the management of hyperthyroidism.
